# Nitroxide pharmaceutical development for age-related degeneration and disease

**DOI:** 10.3389/fgene.2015.00325

**Published:** 2015-11-06

**Authors:** Jacob A. Zarling, Vienna E. Brunt, Anne K. Vallerga, Weixing Li, Albert Tao, David A. Zarling, Christopher T. Minson

**Affiliations:** ^1^Department of Biology, University of Oregon, EugeneOR, USA; ^2^Department of Human Physiology, University of Oregon, EugeneOR, USA; ^3^Colby Pharmaceutical Company, Menlo ParkCA, USA; ^4^Department of Biomedical Engineering, Washington University in St. Louis, St. LouisMO, USA

**Keywords:** aging, inflammation, smoking, CVD, AMD, tempol, hydroxylamine

## Abstract

Nitroxide small molecule agents are in development as preventative or therapeutic pharmaceutical drugs for age-related macular degeneration (AMD) and cardiovascular disease, which are two major diseases of aging. These aging diseases are associated with patient genetics, smoking, diet, oxidative stress, and chronic inflammation. Nitroxide drugs preventing aging-, smoking-, high sugar or high fat diet-, or radiation- and other environmental-induced pathophysiological conditions in aging disease are reviewed. Tempol (TP), Tempol Hydroxylamine (TP-H), and TP-H prodrug (OT-551) are evaluated in (1) non-smokers versus smokers with cutaneous microvascular dysfunction, rapidly reversed by cutaneous TP; (2) elderly cancer patients at risk for radiation-induced skin burns or hair loss, prevented by topical TP; and (3) elderly smoker or non-smoker AMD patients at risk for vision loss, prevented by daily eye drops of OT-551. The human data indicates safety and efficacy for these nitroxide drugs. Both TP and TP-H topically penetrate and function in skin or mucosa, protecting and treating radiation burns and hair loss or smoking-induced cutaneous vascular dysfunction. TP and TP-H do not penetrate the cornea, while OT-551 does effectively penetrate and travels to the back of the eye, preserving visual acuity and preserving normal and low light luminance in dry AMD smokers and non-smoker patients. Topical, oral, or injectable drug formulations are discussed.

## Introduction

Certain age- and environmental-related pathophysiological changes, degenerative conditions, and diseases are driven and accelerated by radical- (i.e., OH, H_2_O_2_, and O^2-^) induced oxidative stress and inflammation (for review, see Wilcox, 2010). For example, chronic exposure to cigarette tobacco smoke can cause and accelerate microvascular dysfunction in cardiovascular disease (CVD) and age-related macular degeneration (AMD). Smoking, together with aging are major causes of diminished vision in AMD, with premature loss of quality of life and independence (Buschini et al., 2015). Radical-induced oxidative stress and inflammation can be modulated by Tempol (TP)-based nitroxide drugs to prevent or treat vascular, ocular, and other pathological conditions and aging associated disease. Pharmaceutical nitroxide drug candidates for treatment and/or prevention of aging-related and smoking-related diseases are in various different stages of development. Clinical data for TP, Tempol Hydroxylamine (TP-H), and the more lipophilic TP-H prodrug, OT-551, are reviewed.

## Pathophysiology of Age-Related Macular Degeneration

Age-related macular degeneration is a major cause of vision loss and loss of independence in the elderly and affects a third of people over 65 (Lim et al., 2012). Dry AMD is a slowly progressing disease involving lipid peroxidation, inflammation, deposition of lipoprotein drusen, and death of photoreceptors, resulting in loss of visual acuity (VA) in both eyes (Buschini et al., 2015). Progression to wet AMD occurs in about 10% of dry AMD patients. Wet AMD is characterized by new blood vessel growth, commonly known as choroidal neovascularization. These new blood vessels leak fluid, causing macular edema, degeneration of retinal tissues, and blindness (Bhutto and Lutty, 2012; Birke et al., 2013).

The etiology of AMD is complex and is based on multiple factors, such as an individual’s genetic susceptibility together with environmental factors and metabolic conditions (Girmens et al., 2012; Buschini et al., 2015). The role of increased oxidative stress is associated with both the incidence and the progression of AMD and is reviewed by Jarrett and Boulton (2012). Both aging and increased oxidative stress mediated diseases are associated with a decrease in transcription factor Nrf2 signaling (Suh et al., 2004; Zhang et al., 2015).

Nrf2, is a major regulator of oxidative stress and regulates the inducible expression of genes encoding phase II detoxification enzymes and anti-oxidant proteins, via the anti-oxidant response element (ARE), to attenuate oxidative stress and protect cell survival (Cano et al., 2010). Nrf2-deficient mice develop retinal pathology that have similarities with human AMD including deregulated autophagy, oxidative injury, and inflammation (Zhao et al., 2011). Wang et al. (2014) demonstrated that Nrf2 is altered in human AMD specimens, and Nrf2 deficiency promotes cellular oxidative damage and a pro-inflammatory environment in cigarette smoke extract (CSE) exposed retinal pigment expthelial (RPE) cells. Human RPE cells exposed to CSE in culture caused oxidative damage and cell death (Bertram et al., 2009). CSE also induced the expression of Nrf2 and exogenous administration of anti-oxidants (GSH and *N*-acetyl-cysteine) prevented oxidative damage to the RPE cells caused by CSE. While Nrf2 signaling has significant cytoprotective capability for RPE cells, aging and/or chronic exposure to a complex and powerful oxidant like cigarette smoke (CS), could impair Nrf2 signaling and promote inflammation sufficient to permit AMD lesion formation (Huang et al., 2015). One logical treatment strategy would be to neutralize oxidative stress.

Primary AMD risk factors include patient genetics, aging, smoking, and diet (Yates and Moore, 2000; Evans, 2001; Tomany et al., 2004; Kifley et al., 2007; Lim et al., 2012; Ersoy et al., 2014). AMD also shares the same risk factors with CVD (Wong, 2010; Cheung and Wong, 2014; Wu et al., 2014). Specific ocular and vascular dysfunction occurs in AMD and CVD, and early CVD diagnoses may indicate early AMD (Cheung and Wong, 2014). Chronic cigarette smoking consistently has a very strong direct positive correlation to AMD. Smoking is also a major modifiable risk factor for CVD, affecting large-vessel atherosclerosis and thrombosis (Bottcher and Falk, 1999). While smoking remains a leading cause of AMD, vascular dysfunction and CVD, it is likely that without effective treatment, several hundreds of millions of people with relatively good vision today, would remain at risk of developing serious vision loss, vascular dysfunction and CVD within relatively few years.

The current treatment option for early dry AMD is life-style change (i.e., smoking cessation, reduced alcohol consumption, controlled diet and increased exercise) together with daily use of anti-oxidant formulations (Buschini et al., 2015). The beneficial use of anti-oxidants in dry AMD was confirmed in the AREDS1 and AREDS2 Phase III clinical studies in which an oral supplement containing anti-oxidants and zinc (AREDS1), and an oral supplement containing anti-oxidants and lutein plus zeaxanthin and/or omega-3 fatty acids (AREDS2) was tested by the National Institutes of Health for halting AMD progression (Age-Related Eye Disease Study 1 Research Group, 2001; Age-Related Eye Disease Study 2 Research Group, 2013). These oral vitamin supplement therapies were shown to modestly retard the progression of dry AMD from an intermediate stage to the advanced stage and the results indicated no overall additional benefit. Current therapeutic approaches in development are reviewed (Girmens et al., 2012; Evans and Syed, 2013; Leung and Landa, 2013; Buschini et al., 2015). Briefly, these therapeutic strategies are to (1) reduce or block the stimulation of continuous damage and inflammation, and/or (2) to replace, repair or regenerate damaged cells.

## OT-551, Tempol Hydroxylamine, and Tempol Drug Development for Age-Related Macular Degeneration

**FIGURE 1:**
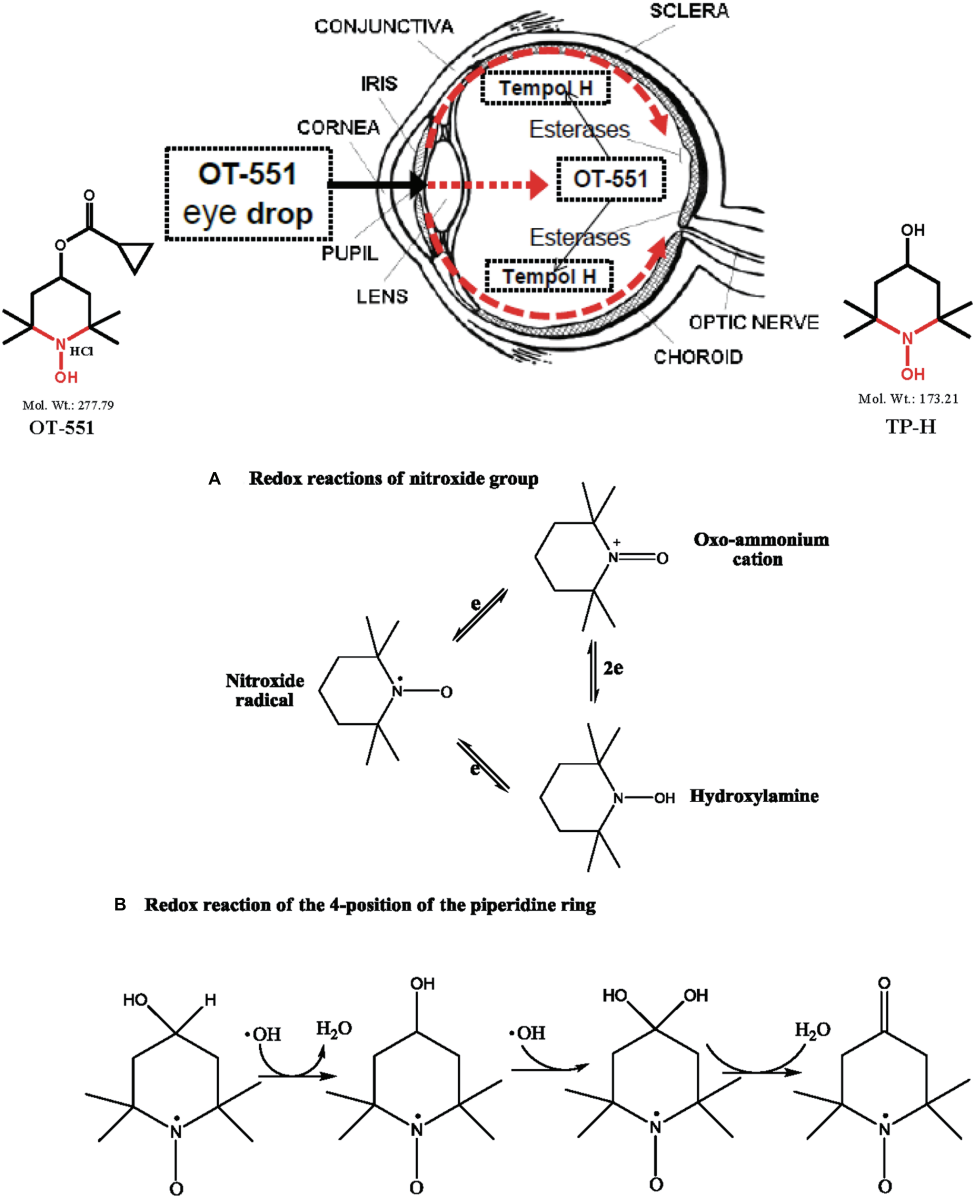
**Piperidine nitroxide OT-551 eye drop for preserving visual acuity (VA) in AMD **(Top)**, nitroxide group redox cycling **(Bottom, A)** and piperidine ring redox reactions (Bottom, B). (Top)** The piperidine nitroxide, OT-551, as an eye drop drug candidate for preserving visual acuity (VA) in AMD **(Top)**. OT-551 is a prodrug of Tempol Hydroxylamine (TP-H; **Top**, middle position). OT-551 is more lipophilic than TP-H and is depicted as a topically administered OT-551 eye drop. The structure of OT-551 is shown (**Top**, far left). OT-551 is self- administered as daily (3× – 4×) eye drops to the front of the human eye of patients with dry AMD (Age related Macular Degeneration), as shown with the thick black arrow. The red dashed arrows indicate the proposed movement of lipophilic OT-551 traveling through the cornea and the scleral route to the back of the eye to the macula, which is the region of greatest VA. OT-551 ester bonds would be cleaved by esterases to yield TP-H (or for short TH, see text) with the chemical structure is shown (**Top**, far right). In the back of the eye the macula can be seen as an oval yellowish colored area surrounding the fovea and near the center of the retina in the area between the two red arrow tips (at about the 3 o’clock position in this eye diagram). **(Bottom, A)** The nitroxide group redox cycling reactions between the nitroxide radical (**A**, left ring), the Oxo-ammonium cation, (**A**, right upper ring), and the Hydroxylamine (**A**, right lower ring). **(Bottom, B)** The piperidine ring redox reactions at the 4-position of the piperidine nitroxide ring.

In *in vitro* screening libraries of small molecules with known activity in inhibiting damage from radicals, TP-H has emerged with desirable safe and strong protective activities against oxidative damage in ocular (Zhou et al., 2008), vascular, and other tissues and organs (Mitchell et al., 2003; Wilcox, 2010). The chemical properties of TP-H, however, prevent it from effectively crossing the cornea (**Figure 1**). As such, topical drug delivery is not feasible for applications to the back of the eye. Therefore, TP-H was chemically modified into a prodrug, OT-551, (with a cyclopropyl group and an ester linkage) which is more lipophilic than TP-H or TP. In contrast to TP and TP-H, OT-551 is fully capable of penetrating the cornea and can travel, via the scleral route, to reach the macula at the back of the eye. In the eye, ocular esterases can convert OT-551 to more water soluble, less lipophilic, TP-H, which can function in the back of the eye, at the macula and retina (**Figure 1**).

OT-551 has been tested for preventative and therapeutic activities in a range of eye, skin, and other conditions. In general, it appears to be a safe and effective small molecule drug candidate which can activate nuclear factor E2-related factor (Nrf-2) and the anti-oxidant response element (ARE), and can inhibit nuclear factor kappa-light-chain-enhancer of activated B cells (NF-κB; Greenwald et al., 2014). As mentioned above, Nrf2 activation modulates a large number of genes, including anti-oxidant enzymes, and genes that control distinct immune and anti-inflammatory responses. Because Nrf2 activation is responsible for cytoprotective functions, nitroxide compounds such as OT-551, TP-H, and TP may be utilized therapeutically to impede smoking-related skin damage and AMD.

OT-551 formulated as a topical daily eye drop for dry AMD patients is capable of significant preservation of both standard luminance VA and low luminance visual acuity (LLVA), which is a measure of impaired night and reduced light vision, as evidenced in human Phase 2 clinical trials for dry AMD. The initial NEI (National Eye Institute, open-label single center) was a National Eye Institute Intramural Research Program sponsored Phase 2 clinical trial for OT-551 in dry AMD which achieved statistical significance for the primary endpoint of preserving VA (Wong et al., 2010). In this NEI trial, the mean change in best corrected visual acuity (BCVA) for the OT-551 study eye was equal to 0.2 ± 13.3 letters, as compared to –11.3 ± 7.6 letters for the fellow control eye (*p* = 0.0259). The NEI trial showed a significant effect of OT-551 on the primary endpoint of VA, but did not show a meaningful effect on the secondary endpoints: growth of Geographic Atrophy (GA) lesion (monitored photographically), drusen size (monitored by optical coherence tomography), or retinal sensitivity (monitored by microperimetry).

In the separate OMEGA (OT-551 Multi Center Evaluation of GA) Phase 2 trial, initiated prior to the completion of the NEI study, the primary endpoint for OT-551 was changed to the prevention of GA, which did not achieve statistical significance. However, in the OMEGA trial, the secondary endpoint for OT-551 was VA, and statistical significance was achieved, as monitored by BCVA and also by LLVA. The month 12 interim OMEGA results showed that the OT-551 positive effect on reducing vision loss at 12 months was statistically significant. OT-551 eye drops at 0.45% significantly (1) reduced loss of VA versus baseline in all study eyes, across all levels of Low Luminance Deficit (LLD; a measure of visual function derived as the difference between BCVA and LLVA; with *p* = 0.0499), and (2) reduced loss in study eyes at higher risk of moderate vision loss with LLD ≥ 15 at baseline (*p* = 0.0360). The month 18 results also showed a positive OT-551 signal. The OT-551 protective trend was maintained at 18 months for LLVA, as monitored by LLD. The administration of 0.45% OT-551, versus the placebo control, reduced loss of vision versus baseline in all study eyes with 18 months data across all levels of LLD.

Thus, the OMEGA results confirmed the NEI results for OT-551 in preserving VA and also extended the data to show OT-551 was capable of preserving LLVA. Direct comparison of the two Phase 2 data sets shows that in the NEI trial the AMD patients had 100% bilateral GA at baseline, while in the OMEGA trial the AMD was less advanced in patients, with only 81% bilateral GA at baseline. Despite this difference in patient cohorts, and differences in the dosing regimen and eye drop size, both NEI and OMEGA trial data suggest that OT-551 eye drops are capable of reducing further loss in VA in AMD patients.

Thus, the OT-551 clinical data from these two different Phase 2 clinical AMD trials indicate that OT-551 appears safe and effective in preserving standard VA and preserving LLVA in dry AMD patients after receiving multiple (3x–4x) daily administrations of eye drops (20–30 μL), as a self-administered topical eye drop formulation. In these clinical studies, the AMD patients’ VA was quantitatively measured in standard luminance or low luminance, via eye chart exams of the OT-551 eye drop instilled eye, as compared to the patients’ randomly chosen control (non-treated) eye. Safety has been demonstrated in over 200 patients who have taken OT-551 for up to 2 years, with no drug-related adverse events. Further studies would be required to determine the effects of OT-551 on VA, LLVA, night vision, inflammation, or inflammatory eye pain in elderly AMD patients who smoke, as compared to non-smoker AMD patients.

The LLD symptoms in intermediate AMD appear to be relevant for the OT-551 OMEGA clinical trial with respect to the important new clinical LLD findings of Wu et al. (2015), suggesting, “VA measures under low luminance conditions may better reflect visual difficulties experienced by individuals. These findings are especially important when considering relevant clinical end points—namely, ‘a characteristic or variable that reflects how a patient feels, functions, or survives,’ as defined by the Biomarkers Definitions Working Group (Atkinson et al., 2001)—for interventional studies targeting the early stages of AMD (see also Csaky et al., 2008; Lesmes et al., 2013).”

There are good reasons to consider that the cigarette smoking status of the OT-551 clinical trial participants may be important and AMD smokers are an appropriate experimental patient group for proposed future dry AMD clinical trials, to directly compare AMD cigarette smoking and AMD non-smoking patients. OT-551 may also be formulated for safe and effective skin or eye protection against smoke- or other chemical damage, as well as for protection against intense white- or especially blue- and ultra-violet-radiation-induced damage, as observed in animal models (Tanito et al., 2007, 2010), or for inflammatory pain. In other human clinical studies, topical administration of TP prevented radiation-induced skin burns and hair loss, when applied to the skin of the head (scalp) of brain cancer patients (Metz et al., 2004).

OT-551 has solid toxicology data (Wilcox, 2010) and is formulated as a topical eye drop for preventing or arresting progression of damage, degeneration, and loss of VA. OT-551 appears safe and effective in dry AMD and has promise for certain other ocular conditions, including in the front of the eye. Lipid peroxidation is an important factor in inflammatory eye conditions, and in progressive and degenerative eye disease (Wilcox, 2010; Tokarz et al., 2013). OT-551 inhibits damage and preserves VA, likely through its various anti-oxidant, anti-inflammatory, anti-angiogenic, or other activities. OT-551’s anti-angiogenic activity does not appear to involve vascular endothelial growth factor (VEGF; a signal protein produced by cells that stimulates vasculogenesis and angiogenesis) inhibition. For AMD patients, this anti-angiogenic activity may potentially be considered as complementary to, or synergistic with, certain approved and marketed AMD anti-angiogenic strategies.

The continued development of OT-551 via immediate or extended release formulations in topical, oral, or parenteral pharma drug products appears warranted for: (a) topical administration as an ophthalmic formulation for aging (and/or smoking) related macular degeneration at the back of the eye, as well as (b) evaluation of OT-551 mediated activities for conditions in the front or inside the eye, including itching and irritation from allergens, smoke, radiation, chemicals, blue-/UV-light and associated inflammatory pain.

## Tempol in Smoking-Related and/or Aging-Related Vascular Conditions and Cardiovascular Disease

Current chronic smoking and advancing age are well-known and accepted risk factors for CVD, including hypertension, coronary heart disease, stroke, and peripheral artery disease (Kannel and Higgins, 1990). Current chronic young or elderly adult smokers have reduced cardiovascular function and smoking causes serious eye, skin, hair, vascular, and other tissue and organ degenerative conditions, including lung, metabolic, and neoplastic diseases. In addition to these diseases and AMD, smoking places individuals at higher risk for many other serious conditions and diseases, many or most of which are related to the cardiovascular system. For example, acute exposure to cigarette smoke causes immediate increases in arterial stiffness and blood pressure (Katayama et al., 2004) as well as reductions in conduit vessel endothelial function (Ijzerman et al., 2003).

The microvasculature is typically the major site of initial vascular damage in CVD progression, as impaired microvascular function can be detected prior to the onset of clinical symptoms (Abularrage et al., 2005; Khan et al., 2005; Joannides et al., 2006). Indeed, it has been well-established that current chronic cigarette smoking significantly impairs microvascular function, even in relatively young, otherwise healthy, individuals (Pellaton et al., 2002; Dalla Vecchia et al., 2004; Edvinsson et al., 2008; Fujii et al., 2013). Furthermore, it has been suggested that impaired microvascular function is also the main mechanism behind other symptoms associated with cigarette smoking, such as increased blood pressure and decreased insulin sensitivity (Attvall et al., 1993).

Much of the risk associated with current chronic smoking and advancing age is related to oxidative stress induced degeneration. Indeed, many cardiovascular conditions and diseases are chronic, progressive reactions initiated, and propagated by local oxidative stress and chronic vascular inflammation (Mangge et al., 2014). Oxidative stress induced inflammation is mediated by different cell types involved in vascular inflammation, producing cytokines with specific membrane receptors for transmission into the cells. Various different cell types communicate, express, and recognize pro-inflammatory or anti-inflammatory cytokines (Ambati et al., 2013). TP-H and TP based agents, may include anti-inflammatory, catalytic anti-oxidant, or anti-angiogenic activities important for various applications in preventative or therapeutic vascular dysfunctions in patients, including those who chronically smoke (**Table 1**). This is probably also true for certain other diet, chemical and radiation exposures in aging-related human conditions of eye, skin, and cardiovascular systems (Hahn et al., 2000; Cheung and Wong, 2014; Greenwald et al., 2014). The skin offers an ideal place to study the therapeutic effects of TP-H or TP formulations on cardiovascular health.

**Table 1:** Human young adult cutaneous microvascular clinical outcomes with cutaneous Tempol (TP).

Reference	Cohort	Presenting	Results	Comments
Fujii et al., 2014	Young adult age current chronic smokers of cigarette tobacco	Impaired microvascular function associated with current chronic cigarette smoking	In 90% of subjects, cutaneous TP administration restored plateau Cutaneous Vascular Conductance, CVC^1^, compared to control non-treated (no TP) cigarette tobacco smokers	TP restores cutaneous microvascular function and Nitric Oxide and Nitric Oxide Synthetase dependent vasodilation
Medow et al., 2011	Young adult age non-smokers (not a cigarette smoker and not exposed to secondhand cigarette smoke)	Healthy; no impaired microvascular function	In 100%, of subjects, cutaneous TP administration had no effect on plateau CVC, compared to controls in young adult non-smokers	Cohort not expected to have high amounts of Reactive Oxygen Species (ROS) and not expected to have impaired microvascular function
Medow et al., 2011	Young adult age non-smokers with oxidative stress experimentally induced by infusion of angiotensin II	Impaired microvascular dysfunction induced by angiotensin II	In 90% of subjects, cutaneous TP administration restored plateau CVC when Reactive Oxygen Species (ROS) was induced by angiotensin II	TP restores microvascular function by inhibiting ROS

*^1^Cutaneous Vascular Conductance (CVC) as evaluated by cutaneous red blood cell flux divided by the mean arterial blood pressure (MAP).*

## Human Studies Showing Mechanisms of Microvascular Dysfunction in Current Chronic Smokers

In general, study of the microcirculation presents a challenge due to difficulties in accurately imaging and reproducibly quantitating microcirculation under minimally or non-invasion conditions. However, the cutaneous microcirculation can be studied relatively easily and non-invasively, and is relevant to smokers and elderly individuals, due to significant skin wrinkle formation, and yellowing of the skin in smokers. Furthermore, the cutaneous microcirculation has been shown to be reflective of generalized microvascular dysfunction in disease progression (Khan et al., 2000; Ijzerman et al., 2003; Kruger et al., 2006). One can study the mechanisms behind impairments in cutaneous microvascular function using techniques such as microdialysis and iontophoresis, in which high concentrations of a vasoactive agent can be delivered to a small area of skin with minimal systemic effects. Using these techniques, test agents can be delivered to both stimulate vasodilation (i.e., acetylcholine) and to inhibit vasodilatory pathways of interest. For example, the role of the nitric oxide pathway can be investigated by administering a nitric oxide synthase inhibitor, such as L-N^G^-nitro-arginine methyl ester (L-NAME).

A number of protocols have been developed to assess cutaneous endothelial function. Two of the most commonly studied are thermal hyperemia and acetylcholine-mediated dilation. The former involves locally heating a small area of skin up to 39–42°C, producing a robust vasodilation, which is primarily dependent on nitric oxide (Kellogg et al., 1999; Minson et al., 2001; Choi et al., 2014). The latter involves infusing acetylcholine into the skin via iontophoresis, microdialysis, or microinjection, which also involves nitric oxide (Kellogg et al., 2005; Medow et al., 2008), albeit to a considerably lesser extent than with thermal hyperemia. Both of these responses are known to be impaired in young smokers (Edvinsson et al., 2008; Fujii et al., 2013, 2014) and with advanced age (Minson et al., 2002; Holowatz et al., 2005).

From studies using these approaches in conscious humans, chronic current cigarette smoking is believed to impair microvascular function primarily by reducing nitric oxide bioavailability via oxidative stress. In support of this notion, Fujii et al. (2013) demonstrated impaired cutaneous microvascular function to be entirely the result of impaired nitric oxide-dependent dilation. Cigarette smoke contains a number of compounds that induce oxidative stress via superoxide production. For example, the semiquinone radical in cigarette tar directly produces superoxide (Pryor and Stone, 1993), and both nicotine (Fang et al., 2006) and stable thiol-reactive agents (Jaimes et al., 2004) activate NADPH oxidase, which produces superoxide. Superoxide combines with nitric oxide to produce hydrogen peroxide, effectively reducing nitric oxide bioavailability and thereby impairing endothelium-dependent dilation.

The mechanisms behind impaired microvascular function in young smokers are remarkably similar to those observed in primary aging. As such, we have proposed that cigarette smoking is a model of premature aging of the vascular system. Primary aging is characterized by reduced nitric oxide bioavailability, secondary to oxidative stress, and much of the vascular impairment can be ameliorated by anti-oxidants, such as ascorbate (Vitamin C), which stoichiometrically scavenges superoxide and also stabilizes tetrabiopterin, an important cofactor for nitric oxide synthase (Heller et al., 2001). For example, intra-arterial infusion of supra-physiological doses of ascorbate in older human subjects improves arterial stiffness (Hildreth et al., 2014) and endothelial function in conduit vessels (Eskurza et al., 2004; Wray et al., 2012) and in the microcirculation of skeletal muscle (Kirby et al., 2009) and the skin (Holowatz et al., 2006). Interestingly, similar effects of ascorbate on conduit-vessel function have been observed in young smokers (Katayama et al., 2004). It is important to note that, in individuals with low levels of oxidative stress (i.e., young, healthy non-smokers), minimal or no effects of certain anti-oxidants have been observed (Eskurza et al., 2004; Kelly et al., 2008).

## Human Clinical Studies with Tempol Measuring Rapid Improvement of Microvascular Function

Fujii et al. (2014) demonstrated TP acutely and rapidly (within 30 min) improves the thermal hyperemia response in young adult smokers, returning the response back to that typically observed in healthy non-smokers and effectively reversing their impaired endothelial function observed. This effect was found to be entirely nitric oxide-dependent. As such, the authors concluded the effect of TP was due to a removal of oxidative stress, and therefore increased nitric oxide bioavailability (Fujii et al., 2014). The various TP-based nitroxide drug candidates which can be best used to improve cutaneous microvascular function or reduce the cardiovascular burden of cigarette smoking in humans remains to be systematically investigated.

Tempol based piperidine nitroxides may have similar effects in the aging microvasculature. Medow et al. (2011) observed TP could fully reverse the reduction in thermal hyperemia caused by infusion of angiotensin-II in young adults. Angiotensin-II is elevated with advanced age, as well as in many disease states, and induces oxidative stress by activating NADPH oxidase and xanthine oxidase. Thus, infusion of angiotensin-II mimics an aging state. Furthermore, DuPont et al. (2014) demonstrated both TP and Apocynin, an inhibitor of NADPH oxidase, ameliorate the impaired thermal hyperemia observed in chronic kidney disease, another disease state characterized by high oxidative stress.

Although no studies to date have administered TP systemically in humans to treat endothelial dysfunction in other microvascular beds (i.e., in the heart, kidneys, etc.), experimental animal studies have shown systemic TP to be effective for improving microvascular function under conditions of high oxidative stress (Wilcox, 2010). For example, chronic administration of TP improved skeletal muscle microvascular function in obese rats (Frisbee, 2005) and retinal microvascular function in diabetic mice (Yadav and Harris, 2011). Vascular impairments associated with angiotensin-II administration were also prevented in mesenteric arteries of rats treated with TP for 2 weeks (Wang et al., 2010).

## Conclusion and Future Directions

These nitroxide agents appear to be safe and effective drugs with anti-oxidant and other activities. They inhibit tissue oxidative stress and inflammation, and also have very significant effects on the gut microbiome, food energy utilization, and metabolism (Li et al., 2013; Kim et al., 2015). TP and TP-H, as piperidine nitroxides, have properties which mimic superoxide dismutase enzyme (SOD) activity. The membrane permeability and potency of small molecule TP, TP-H, OT-551, and analogs can be relatively superior to SOD as a human enzyme. TP has a relatively high rate constant in catalytic decompositions of peroxynitrate-derived free radicals, including nitrogen dioxide, carbonate, and superoxide as free radicals. Peroxynitrate is produced in the reaction of nitric oxide with superoxide anions in vasculature, ocular, skin, and other tissues. Nitroxide based drugs, like TP-H or TP, can protect against peroxynitrate damage in red and other blood cells, platelets, and blood plasma, as well as protect proteins and lipids in other cells and tissues.

In summary, there are promising uses of these nitroxide-based drugs as anti-inflammatory, anti-angiogenic, or anti-oxidant agents, in reversing impaired cutaneous endothelial function for smokers; preserving VA in AMD; or inhibiting disease conditions where inflammation, angiogenesis, or oxidative stress are mediators of dysfunction. Future studies are warranted to determine whether TP, TP-H, OT-551 prodrug, or analogs can be used safely and effectively in topical, oral, or injectable formulations to promote health, by preventing or therapeutically treating certain chronic inflammatory conditions or aging-related degeneration and disease.

## Conflict of Interest Statement

The authors declare that the research was conducted in the absence of any commercial or financial relationships that could be construed as a potential conflict of interest.
